# Clinical value of preventive balloon dilatation for esophageal stricture

**DOI:** 10.3892/etm.2012.753

**Published:** 2012-10-19

**Authors:** CHANGXIONG WANG, XIANGHONG LU, PING CHEN

**Affiliations:** Digest Endoscope Center, People’s Hospital of Lishui, The Sixth Affiliated Hospital of Wenzhou Medical College, Lishui, Zhejiang 323000, P.R. China

**Keywords:** esophageal diseases, endoscopic therapy, balloon dilatation, prevention

## Abstract

Endoscopic mucosal resection (EMR) and endoscopic submucosal dissection (ESD) were developed for the treatment of benign lesions and early superficial esophageal cancers in the gastrointestinal (GI) tract. However, esophageal strictures frequently develop in patients who undergo EMR/ESD. Therefore, we aimed to investigate the clinical value of preventive balloon dilatation (BD) for esophageal diseases following endoscopic therapy. A total of 30 patients who had received EMR or ESD were enrolled in the study. Preventive BD was carried out for 12 cases within 1 week following EMR/ESD. The remaining 18 cases were not subjected to preventive BD and were used as an historic control. The results revealed that no complications, including esophageal stenosis and dysphagia, were observed in the patients who received preventive BD. In the control group, seven cases experienced dysphagia, of which two were released without clinical treatment and the other five were released following two or three BD procedures. The results indicate that preventive BD is an effective treatment for patients with esophageal diseases following EMR and should be considered at an early stage when the mucosal injury exceeds two-thirds of the esophageal lumen.

## Introduction

Endoscopic mucosal resection (EMR) and endoscopic submucosal dissection (ESD) were developed for the treatment of benign lesions and early superficial esophageal cancers in the gastrointestinal (GI) tract (1). EMR is typically used for the removal of lesions smaller than 2 cm or for the piecemeal removal of larger lesions. For the removal of larger lesions, ESD is usually required (1). EMR has been established as a treatment for superficial esophageal cancer due to its minimal invasiveness and excellent survival rate (2,3) and ESD enables the resection of widespread neoplasia, including Barrett’s esophageal cancer (4,5). However, esophageal strictures frequently develop in patients who undergo EMR/ESD (6). Esophageal stricture may seriously interfere with the oral intake of food and fluids, and thus lead to a decline in patients’ quality of life. More significantly, once severe esophageal stricture has developed, it is difficult to reverse the condition.

A number of methods have been reported to prevent esophageal stricture following EMR/ESD (7–9). Among them, balloon dilatation (BD) has been indicated as a frequently used technique for alleviating esophageal strictures post EMR/ESD. A retrospective study revealed that preventive BD reduced the incidence of esophageal stricture in patients who underwent an extensive EMR/ESD (10). However, the efficacy of preventive BD requires further confirmation. In the present study, we carried out a prospective randomized trial between September 2008 and September 2011 on 30 cases who received EMR/ESD treatment in our hospital.

## Patients and methods

### Patients

Between September 2008 and September 2011, 30 patients, including 9 cases with postoperative benign tumors and 21 cases with postoperative early-stage mucosal lesions, were enrolled in the study (Table I). Twelve patients received preventive BD 2 weeks after EMR/ESD therapy and 18 patients who had undergone EMR/ESD treatment served as a control. Written informed consent was obtained from all patients prior to carrying out EMR/ESD and BD. The study was approved by the Ethics Committees of the People’s Hospital of Lishui.

### Treatment protocol

**Figure 1:**
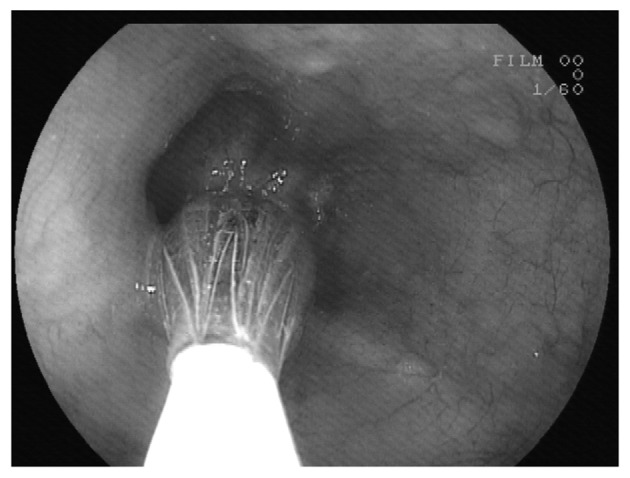
Wound area at the middle of the balloon.

**Figure 2:**
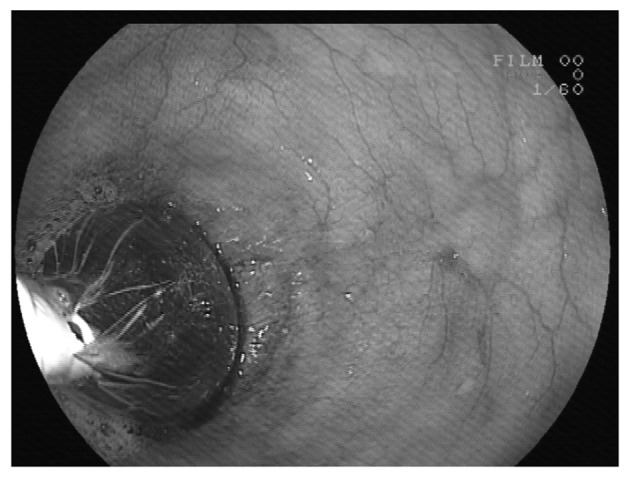
Light bleeding at the wound focus.

**Figure 3:**
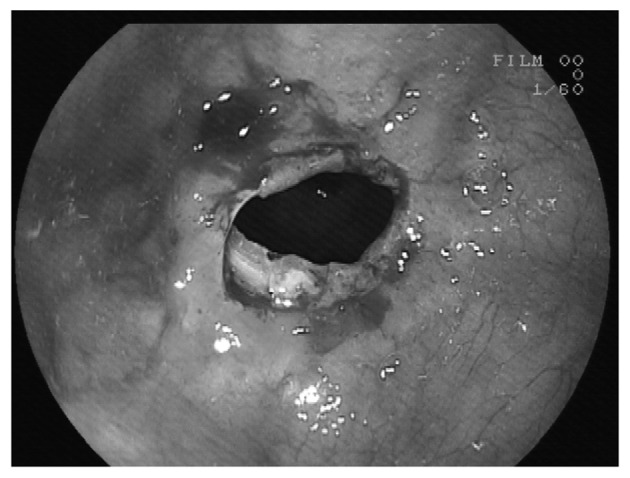
Postoperative carotid dilatation.

EMR/ESD treatment was performed according to previously described methods (11,12). The patients in the control group underwent common endoscopic re-examination. For patients receiving preventive BD, routine blood tests, clotting and bleeding time tests and an electrocardiogram (ECG) were performed prior to the treatment. Following 8 h of fasting, 0.5 mg atropine and 10 mg diazepam were injected intramuscularly. The local area was anesthetized with lidocaine mortar and the lubricated balloon catheter was inserted. The balloon was positioned at the center of the primary lesion and then inflated carefully with air to reach a pressure of 6 psi under the control of a pressure pump (Fig. 1). This dilatation was maintained for 1–3 min according to the patient’s situation, and the wound was monitored for bleeding. If bleeding occurred, it suggested that the dilatation was efficient (Fig. 2). The air was then released, the dilatation was repeated 3 min later and the balloon was then removed. Gastroscopy was used to observe the extent of the expansion of the stenosis and the possible complications (Fig. 3). During the procedure, the pulse, blood pressure and oxygen saturation of the patient were closely observed.

## Results

### Efficacy of preventive BD

In the treatment group, every patient was treated with preventive BD only once and the result was observed to be satisfactory. One year follow-up revealed that no complications, including esophageal stenosis, dysphagia, esophageal perforation, heavy bleeding or mortality, had occurred. However, in the control group, seven cases experienced dysphagia, of which two were released without clinical treatment and the other five were released following two or three BD procedures (Table II).

## Discussion

EMR and ESD are efficient endoscopic techniques for minimally invasive therapy. However, the occurrence rate of cicatricial stenosis of postoperative esophageal diseases is 6–26% (13). Stenosis of the esophagus seriously affects the patients’ quality of life and causes certain complications, including poor nutrition. Dilatation at an early stage may alleviate the severity of the stenosis and, to some extent, avoid the formation of permanent cicatricial stenosis at later stages which may result in esophageal reconstruction due to dysphagia. BD using endoscopic guidance has been demonstrated to be very efficient for the treatment of esophageal stenosis (14). The balloon is able to apply pressure uniformly all around the esophagus, leading to the breakdown of fibrous scar tissue bundles around the stenosis and even the breakdown of muscle fibers and the subsequent relaxation of the esophageal lumen (1). BD therapy has the advantages of uniform pressure, being doubly controlled by the endoscope and the sensations of the patients. With these advantages, it is easy to control the extent of dilatation, avoid complications and obtain a clear field to increase the safety and success rate. Therefore, BD has been considered as the most favorable method for the treatment of esophageal stenosis and as a substitute for surgery (1,15). In the present study, no esophageal stenosis occurred in the patients treated with preventive BD in the one year follow-up period, whereas in the control group, five cases reported serious dysphagia and were treated several times with BD, indicating that preventive BD was able to prevent long-term complications in patients undergoing EMR/ESD. The current study indicates that patients with a wound area affecting over two-thirds of the esophageal lumen following EMR/ESD for esophageal diseases should receive preventive BD at an early stage to avoid esophageal stenosis and improve the quality of life.

In conclusion, the current study indicates that preventive BD may be considered as an effective therapy to reduce the incidence of esophageal stenosis following EMR/ESD. Since there is no other effective method for avoiding esophageal stenosis following EMR/ESD at present, preventive BD should be considered for all patients who undergo EMR/ESD. However, there are also certain shortcomings of the present study. Firstly, the number of patients is rather small, which limits the credibility of the study. Secondly, the follow-up period of the patients is only one year and certain complications may not have yet arisen. Therefore, further studies that include more patients and are maintained for a longer period are required to confirm the effectiveness of preventive BD for the prevention of esophageal stenosis following EMR/ESD in patients with early stage esophageal cancer.

## Figures and Tables

**Table I:** Patients’ backgrounds.

	Gender		Diseases		Therapy	
Group	Male	Female	Benign tumor	Early lesion	EMR	ESD
Control	9	9	5	13	5	13
Treatment	6	6	4	8	3	9

EMR, endoscopic mucosal resection; ESD, endoscopic submucosal dissection.

**Table II:** Patients’ conditions following EMR/ESD.

		Dysphagia			
Group	Preventive BD	Transient	Persistent	Dilatation	Complications
Control	0	2	5	5	0
Treatment	12	0	0	0	0

EMR, endoscopic mucosal resection; ESD, endoscopic submucosal dissection; BD, balloon dilatation.
